# Joint Optimization of Data Freshness and Fidelity for Selection Combining-Based Transmissions

**DOI:** 10.3390/e24020200

**Published:** 2022-01-28

**Authors:** Zhengchuan Chen, Mingjun Xu, Min Wang, Yunjian Jia

**Affiliations:** 1 School of Microelectronics and Communication Engineering, Chongqing University, Chongqing 400044, China; xmj@cqu.edu.cn; 2 State Key Laboratory of Integrated Services Networks, Xidian University, Xi’an 710071, China; 3 School of Optoelectronics Engineering, Chongqing University of Posts and Telecommunications, Chongqing 400065, China; wangm@cqupt.edu.cn

**Keywords:** wireless sensor network, age of information, sensor node deployment

## Abstract

Motivated by big data applications in the Internet of Things (IoT), abundant information arrives at the fusion center (FC) waiting to be processed. It is of great significance to ensure data freshness and fidelity simultaneously. We consider a wireless sensor network (WSN) where several sensor nodes observe one metric and then transmit the observations to the FC using a selection combining (SC) scheme. We adopt the age of information (AoI) and minimum mean square error (MMSE) metrics to measure the data freshness and fidelity, respectively. Explicit expressions of average AoI and MMSE are derived. After that, we jointly optimize the two metrics by adjusting the number of sensor nodes. A closed-form sub-optimal number of sensor nodes is proposed to achieve the best freshness and fidelity tradeoff with negligible errors. Numerical results show that using the proposed node number designs can effectively improve the freshness and fidelity of the transmitted data.

## 1. Introduction

With the emergence of the Internet of Things (IoT) and continuous development of communication techniques, increasingly more facilities are connected to the Internet. At the same time, a flood of information swarms into the fusion center (FC) waiting to be processed.

Wireless sensor network (WSN) is a typical data-driven application in IoT, where multiple sensor nodes are deployed dispersedly aiming at observing the information source. The observed information is then transmitted through wireless channels, reaching the FC for further data gathering and recovery. Typical examples of WSN include forest salinity monitoring, smart parking, intelligent transportation safety supervising, and so on.

In WSN, the freshness of the data is essential for taking prompt actions. For example, in a pedestrian detection system, timely data from sensors in-vehicle and infrastructure is critical to avoid collision and ensure the safety of pedestrians. In smart parking, the occupancy of the parking lot is vital for the users. In environment supervising, fresh data from the sensors is urgently wanted to better monitor the air pollution condition [1]. The above examples show the significance of the freshness of the received information in wireless sensor networks since it is transmitted to operate, supervise, and monitor the systems.

Aside from the freshness, ensuring the fidelity, or accuracy of the received data is also of paramount importance. In wireless communication systems, diversity techniques are applied as useful tools for combating fading effects and improving the quality of the received signal [2,3,4,5,6]. The fundamental diversity combining is the selection combining (SC) scheme, where there is a signal to noise ratio (SNR)-based SC scheme [3] and log-likelihood ratio (LLR)-based SC scheme [5]. For the LLR-based SC scheme, it selects the branch based on the LLR. For SNR-based SC scheme, it selects the branch that provides the largest SNR from several diverse branches. This approach can effectively improve the fidelity of the ultimately received data.

Based on the background information, we can conclude that when accounting for these two metrics, on the one hand, better freshness can be achieved by waiting for observations from fewer sensor nodes, yet it leads to a decline in the fidelity as there might be insufficient observations; On the other hand, a higher fidelity of transmitted data can be obtained when FC receives more observations from sensors. However, the data may become stale since there may be a long time for receiving enough observations. Motivated by the opposite trend of two metrics affected by the number of sensor nodes, we aim to find the optimal number of sensor nodes by carefully characterizing the data freshness and fidelity using the age of information (AoI) and minimum mean square error (MMSE) metric.

### 1.1. Contributions

Contributions of this paper can be summarized as follows.

1. We establish a theoretical model that simultaneously takes into account the data freshness and fidelity for jointly optimizing two metrics through adjusting the number of sensor nodes. Explicit characterizations of freshness and fidelity were given by applying the average AoI and MMSE metrics.
2. By endowing the weighting factor to the average AoI and MMSE, the optimal number of sensor nodes optimization problem is formulated. Through appropriate approximations on both metrics, we specify the optimal number of sensor nodes by proposing a sub-optimal solution, which jointly minimizes data freshness and fidelity.
3. Numerical results validate the correctness of the proposed solution and it can achieve the best freshness-fidelity weighted-sum tradeoff with negligible errors.

### 1.2. Organizations

The remainder of this paper is organized as follows. The related works are demonstrated in Section 2. In Section 3, we introduce the system model. In Section 4, we formulate the problem by introducing the freshness and fidelity metric. Closed-form sub-optimal solution is given based on appropriate approximations in Section 5. Numerical results are provided in Section 6. Finally, conclusions are drawn in Section 7.

## 2. Related Works

In this Section, we provide a thorough overview of the current academic and state-of-the-art works, to provide a broader perspective and deeper understanding of our work.

In 2011, a metric termed the age of information (AoI) was widely applied to characterize the freshness of data delivered in a communication system. It is defined as the time elapsed since the generation of the latest received update [7]. Various systems characterized by different queueing models such as M/M/1, D/M/1, M/D/1, M/G/1, and G/G/1/1 adopted AoI to evaluate the freshness of the received data [7,8,9]. Authors in [10] considered the multi-source preemptive queuing model and investigated the optimal generation rate of each source achieving the best information freshness. The optimal generation rate of update which induced the minimal violation probability is found in [11]. Apart from the average AoI metric, The metric of peak AoI (PAoI) was introduced to capture information about the maximum value of the AoI [12,13]. Based on the violation probabilities of AoI and peak AoI, the authors in [14] analyzed the optimal arrival rate of status update in the sight of asymptotical optimality. Authors in [15] proposed a new metric termed age upon decisions (AuD) to evaluate the freshness of received updates at some decision epochs.

In WSN, the mean square error metric is widely applied to characterize the fidelity of the system. In [16], techniques for designing robust pre-coders and combiners to the linear decentralized estimation of unknown vector parameters in a coherent multiple-input multiple-output (MIMO) network with multiple sensors under imperfect channel state information (CSI) were presented. Their proposed techniques were based on the criteria that minimize the MSE of the estimated signal at the FC limited by total network power constraint. The authors in [17] proposed a framework of joint collaboration-compression for sequential estimation of a random vector parameter in WSN. By alternatively minimizing the sequential MMSE, they designed near-optimal and linear compression strategies under power constraints. Following from [16], the authors continued to develop optimal pre-coders minimizing the sum-MSE for the scenario of transmitting quantized observations in [18].

There have been typical works focusing on freshness and fidelity metrics in WSN [19,20,21,22]. An adaptive monitoring framework was introduced in [19] to achieve a balance between efficiency and accuracy on Internet-enabled physical devices. The authors in [20] determined the age-optimal policies for the update request and processing times subject to a maximum allowed distortion constraint on the updates. In [21], it was proved that there is an optimal number of quantization bits that optimizes the tradeoff of AoI and the MMSE. In [22], the AoI and MSE metrics were explicitly characterized by deriving the closed-form expressions for the block lengths and accuracy levels, after which they optimized the coding schemes by demonstrating a reachable region of AoI and MSE.

The above-mentioned works focused on the impact of request time, quantization bits, and the block length, respectively, on system freshness and fidelity. While our work is distinguished from them in that we try to optimize system performance through adjusting the number of sensor nodes, which is of paramount significance at the very beginning deploying phase.

## 3. System Model

**Figure 1:**
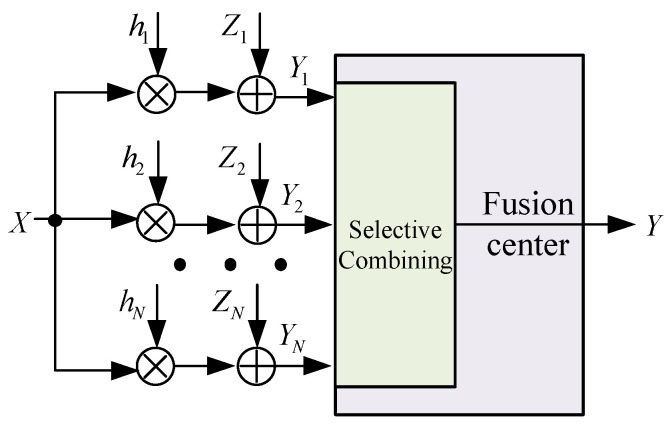
Signal model of the proposed system.

We consider a wireless sensor network consisting of *N*, N∈ℕ sensor nodes, which observe a common metric *X* (state update information such as temperature, humidity, and speed) characterized by a Gaussian process with zero-mean and variance $\sigma_{X}^{2}$ (the assumption is based on the pervasiveness that a large number of phenomena are distributed in normal form, as is assumed in [23,24,25]), as illustrated in Figure 1. Each node’s observation is transmitted through an orthogonal channel, which follows block Rayleigh fading. For ease of manipulation, we assume that the observation experience independent channel gain denoted as $|h_{i}{|}^{2}∼\exp\left(1\right)$, channel noise $Z_{i}$ with zero-mean and variance $\sigma_{Z_{i}}^{2}$. The received signal for the *i*-th node $Y_{i}$ is expressed as:

(1) $$\begin{array}{c} Y_{i}=h_{i}X+Z_{i}, \end{array}$$

where $h_{i}$ can be obtained by transmitting pilot signal which is used for channel estimation.

Then, the observations are transmitted to the FC. Considering that the ability of signal processing, collecting, and transmission are different among the facilities, we assume the transmitting time from sensor nodes to the FC obeys exponential distribution with mean 1/v and is independent and identically distributed (i.i.d.) across the sensor nodes. Specifically, the transmission is organized in rounds where the FC waits for observations from *N* sensors in each round, and it begins a new round at the exact moment when the previous round is complete. At the FC, selection combining (SC) technology is employed to better control the fidelity of the received data.

### 3.1. Data Freshness Metric

We adopt the AoI of the FC as the freshness metric of the system. It is defined as the difference between the current time and the time when the most recently received observation is generated. Formally, at a given time t, AoI is expressed as:

(2) $$\Delta_{t}:=t-u_{t},$$

where $u_{t}$ represents the generating time of the most recently received observation. Accordingly, as is described in [7], the average AoI in the interval of (0,T) is given as:

(3) $$\bar{\Delta }=\lim_{T\to \infty }\frac{1}{T}\int_{0}^{T}\Delta_{t}\;dt.$$

**Figure 2:**
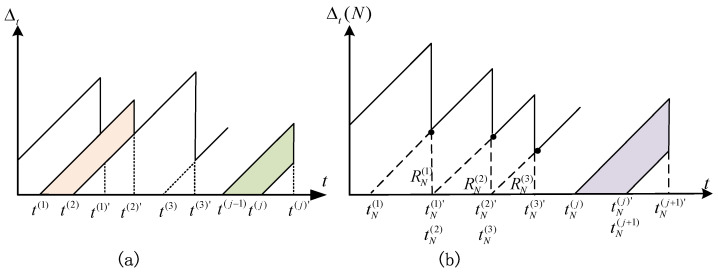
(**a**) Typical AoI evolution. (**b**) AoI evolution of our proposed system.

Figure 2a gives a typical example of the AoI evolution curve where $t^{\left(1\right)}$ represents the generation time of the first update, and $t^{{\left(1\right)}^{\prime }}$ is the time it arrives at the FC. The AoI curve keeps growing until a new update is received by the FC, thus it exhibits a sawtooth shape. However, recall that in our proposed model, the FC works in rounds (round index shown in the form of superscript), where, in each round, there are *N* sensors(shown in the form of subscript) observing the source, and a new round of data collecting begins at the exact moment when the previous round is finished, i.e., $t_{N}^{{\left(1\right)}^{\prime }}=t_{N}^{\left(2\right)}$, etc. As shown in Figure 2b, the average AoI can be obtained through calculating the difference between the isosceles triangle expressed as $\frac{1}{2}[{\left(R_{N}^{\left(j\right)}+R_{N}^{\left(j+1\right)}\right)}^{2}-{\left(R_{N}^{\left(j+1\right)}\right)}^{2}]$. Specifically, considering *I* rounds of sensing in total, the time average AoI is:

$$\begin{array}{cc} \bar{\Delta } & =\lim_{I\to \infty }\frac{\sum_{j=1}^{I}[\frac{1}{2}{\left[R_{N}^{\left(j\right)}\right]}^{2}+R_{N}^{\left(j\right)}R_{N}^{\left(j+1\right)}]}{\sum_{j=1}^{I}R_{N}^{\left(j\right)}}=\frac{𝔼\left[{\left(R_{N}^{\left(j\right)}\right)}^{2}\right]+2𝔼\left[R_{N}^{\left(j\right)}\right]𝔼\left[R_{N}^{\left(j+1\right)}\right]}{2𝔼[R_{N}^{\left(j\right)}]}. \end{array}$$

where 𝔼[·] represents the mathematical expectation operator. Note that $𝔼\left[R_{N}^{\left(j\right)}\right]$ and $𝔼\left[R_{N}^{\left(j+1\right)}\right]$ are independent from each other as round index approaches infinity, we have $𝔼\left[R_{N}^{\left(j\right)}\right]𝔼\left[R_{N}^{\left(j+1\right)}\right]=𝔼{\left[R_{N}^{\left(j\right)}\right]}^{2}$. Notice that the statistical characteristics of $R_{N}^{\left(j\right)}$ are the same, and $R_{N}^{\left(j\right)}$ is actually related to the number of sensor nodes *N*, so we remove the superscript *j* and specify the number of sensor nodes *N* as a subscript of the interval random variable, i.e., $R_{N}$. Then, the average AoI is

(4) $$\begin{array}{cc} \bar{\Delta }\left(N\right) & =𝔼\left[R_{N}\right]+\frac{𝔼[R_{N}^{2}]}{2𝔼[R_{N}]}. \end{array}$$

### 3.2. Data Fidelity Metric

We use selection combining technology at the FC, i.e., we select the largest $|h_{i}|$ among all sensor nodes denoted as $|h_{\max}|$, i.e., $\max\{|h_{i}\left|,1\leq i\leq N\}=\right|h_{\max}|$. And that results in:

(5) $$\begin{array}{c} Y=|h_{\max}|X+Z_{i}. \end{array}$$

Accordingly, the distortion between the source *X* and the received signal *Y* is denoted as *D*, which is expressed as

(6) $$\begin{array}{cc} D & =𝔼{\left[kY-X\right]}^{2}=𝔼{\left[\left(k\right|h_{\max}\left|-1\right)X+kZ_{i}\right]}^{2}, \end{array}$$

where *k* is the signal estimating coefficient.

## 4. Problem Formulation

In this section, we firstly give explicit characterizations of two metrics. After that, a problem of jointly optimizing data freshness and fidelity of the sensing system is formulated.

### 4.1. Average AoI of the Proposed Model

**Lemma** **1.** *The average AoI observed at the FC is:*

(7) $$\begin{array}{c} \bar{\Delta }\left(N\right)=\frac{3H_{N}}{2v}+\frac{G_{N}}{2vH_{N}}, \end{array}$$

*where $H_{N}=\sum_{l=1}^{N}\frac{1}{l}$ and $G_{N}=\sum_{l=1}^{N}\frac{1}{l^{2}}$.*

**Proof of Lemma 1.**  Recall that the general expression on average AoI we derive in Equation (4) requires us to figure out $𝔼[R_{N}]$ and $𝔼[R_{N}^{2}]$. And the transmitting time from sensor nodes to the FC obeys exponential distribution with mean 1/v and is i.i.d. across the sensor nodes. In terms of order statistic [26], the mathematical expectation and variance of the time difference denoted as 𝔻[·] between the first arriving observation and the start point is given by:

(8) $$\begin{array}{c} 𝔼\left[R_{1}-R_{0}\right]=\frac{1}{Nv},\;𝔻\left[R_{1}-R_{0}\right]=\frac{1}{N^{2}v^{2}}, \end{array}$$

as we treat this observation as the first arriving observation among *N* sensors. As we set $𝔼[R_{0}]$ and $𝔻[R_{0}]$ to 0, $𝔼[R_{1}]$ and $𝔻[R_{1}]$ can be calculated as:

(9) $$\begin{array}{c} 𝔼\left[R_{1}\right]=\frac{1}{Nv},\;𝔻\left[R_{1}\right]=\frac{1}{N^{2}v^{2}}. \end{array}$$

The mathematical expectation and variance of the time difference between the second and first observation is:

(10) $$\begin{array}{c} 𝔼\left[R_{2}-R_{1}\right]=\frac{1}{(N-1)v},\;𝔻\left[R_{2}-R_{1}\right]=\frac{1}{{\left(N-1\right)}^{2}v^{2}}, \end{array}$$

as we treat this observation as the first arriving observation among the remaining N−1 sensors. Take Equation (9) into Equation (10), $𝔼[R_{2}]$ and $𝔻[R_{2}]$ can be figured out as:

$$\begin{array}{cc} & 𝔼\left[R_{2}\right]=\frac{1}{Nv}+\frac{1}{(N-1)v}=\frac{H_{N}-H_{N-2}}{v},\\ & 𝔻\left[R_{2}\right]=\frac{1}{N^{2}v^{2}}+\frac{1}{{\left(N-1\right)}^{2}v^{2}}=\frac{G_{N}-G_{N-2}}{v^{2}}. \end{array}$$

By using Mathematical induction, the *N*-th arriving observation is given by:

(11) $$\begin{array}{c} 𝔼\left[R_{N}\right]=\frac{H_{N}}{v},\;𝔻\left[R_{N}\right]=\frac{G_{N}}{v^{2}}. \end{array}$$

Note that $𝔼[R_{N}^{2}]$ can be expressed as

(12) $$\begin{array}{cc} 𝔼[R_{N}^{2}] & ={𝔼}^{2}\left[R_{N}\right]+𝔻\left[R_{N}\right]. \end{array}$$

By taking Equations (11) and (12) into Equation (4), the proof is completed. □

### 4.2. MMSE of the Proposed Model

Recall that in our model each node transmits its observation through block Rayleigh fading channel, where the channel gain within a block remains the same while it varies independently across different blocks. Based on this model, let us use the MMSE metric to evaluate the data fidelity of the system.

**Lemma** **2.** *The MMSE $\bar{D}\left(N\right)$ by using SC technique is:*

(13) $$\begin{array}{c} \bar{D}\left(N\right)=\sum_{i=1}^{N}{\left(-1\right)}^{i-1}\left(\frac{i}{N}\right)i\Gamma \left(0,\frac{i}{\gamma_{0}}\right)e^{i/\gamma_{0}}, \end{array}$$

*where $\Gamma \left(a,b\right)=\int_{b}^{\infty }u^{a-1}e^{-u}du$ represents the incomplete Gamma function.*

**Proof of Lemma 2.**  Firstly, one can establish objective problem of minimizing the distortion as shown in Equation (6), which is

(14) $$\begin{array}{c} \min_{k}\;𝔼{\left[\left(k\right|h_{\max}\left|-1\right)X+kZ_{i}\right]}^{2}, \end{array}$$

where $𝔼{\left[X\right]}^{2}=\sigma_{X}^{2}$ and $𝔼{\left[Z_{i}\right]}^{2}=\sigma_{Z_{i}}^{2}$. Notice the irrelevance between source *X* and the noise $Z_{i}$, the objective problem can be further elaborated as

(15) $$\begin{array}{c} \min_{k}\;\left(\right|h_{\max}{|}^{2}\sigma_{X}^{2}+\sigma_{Z_{i}}^{2})k^{2}-2\left|h_{\max}\right|\sigma_{X}^{2}k+\sigma_{X}^{2}. \end{array}$$

With some manipulations, the distortion, i.e., the optimal value of Equation (15) can be written as

(16) $$\begin{array}{c} D\left(N\right)=\frac{\sigma_{X}^{2}\sigma_{Z_{i}}^{2}}{|h_{\max}{|}^{2}\sigma_{X}^{2}+\sigma_{Z_{i}}^{2}}. \end{array}$$

In our model, we assume the noise power is the same among all the sensor nodes, i.e., $\sigma_{Z_{i}}^{2}=\sigma_{Z}^{2}=1$. Denote the SNR of each node as $\gamma_{0}=\sigma_{X}^{2}/\sigma_{Z}^{2}$, then the distortion is

(17) $$\begin{array}{c} D\left(N\right)=\frac{\gamma_{0}}{|h_{\max}{|}^{2}\gamma_{0}+1}=\frac{1}{|h_{\max}{|}^{2}+\frac{1}{\gamma_{0}}}. \end{array}$$

Recall $|h_{i}{|}^{2}∼\exp\left(1\right)$ and $\max\{|h_{i}\left|,\;1\leq i\leq N\}\;=\;\right|h_{\max}|$. Now we need to figure out the probability density function (pdf) of $|h_{\max}{|}^{2}$ to calculate the MMSE. For conciseness we denote $|h_{\max}{|}^{2}$ and its corresponding pdf as *g* and p(g), respectively. Given *N* i.i.d. exponential distribution random variables with parameter 1/θ, the pdf of the maximum variable among them can be derived:

(18) p(g)=Nθe−g/θ(1−e−g/θ)N−1=Nθe−g/θ∑i=0N−1iN−1(−e−g/θ)i=∑i=0N−1(−1)iNiN−11θe−g(i+1)/θ=∑i=1N(−1)i−1iNiθe−gi/θ

where in our work we have 1/θ=1. After that, the MMSE can be computed as:

D¯(N)=∫0∞1g+1γ0p(g)dg=(a)∑i=1N(−1)i−1iNi∫iγ0∞1ωe−ωdωei/γ0=∑i=1N(−1)i−1iNiΓ(0,iγ0)ei/γ0,

where (a) follows from $\omega =(g+\frac{1}{\gamma_{0}})i$ and $d\omega =idg+\frac{i}{\gamma_{0}}$. □

### 4.3. Tradeoff between Data Freshness and Fidelity

Following the analyses in the above two subsections, we can see that the number of sensor nodes *N* is essential in affecting both freshness and fidelity metrics. Based on the results we derive so far, the objective problem is formulated by importing a weighting factor α, to jointly optimize the two metrics simultaneously through adjusting the number of sensor nodes and the weighting factor:

(19) $$\begin{array}{ccc} {𝒫}_{1}:\min_{N\in \mathbb{N}} & f\left(N\right)=\alpha \bar{\Delta }\left(N\right)+\left(1-\alpha \right)\bar{D}\left(N\right), & \end{array}$$

where $\bar{\Delta }\left(N\right)$ and $\bar{D}\left(N\right)$ are given by Equations (7) and (13).

We can see that when the weighting factor α is large, the average AoI part is dominant. This condition can be applied in scenarios such as daily news updating and music hits ranking where timeliness is more important. On the contrary, when α is relatively small, the fidelity of the system becomes the major concern while the freshness requirement becomes relatively low. Such systems like fuel consumption reporting and indoor temperature monitoring are in this case since the state of these systems often changes slowly over time.

In order to find the optimal $N^{🟉}$, we first analyze the monotonicity of $\bar{\Delta }\left(N\right)$, $\bar{D}\left(N\right)$ and the tradeoff f(N) respectively.

#### 4.3.1. Monotonicity of Average AoI

As is derived from Lemma 1, the monotonicity of Δ(N) can be figured out by calculating the difference between Δ(N) and Δ(N−1):

(20) $$\begin{array}{cc} \bar{\Delta }\left(N\right)-\bar{\Delta }\left(N-1\right) & =\frac{3N+H_{N-1}-NG_{N-1}}{2vN^{2}}. \end{array}$$

It can be easily verified that Equation (20) is positive, which indicates $\bar{\Delta }\left(N\right)$ monotonically increases w.r.t. the number of sensor nodes *N*.

#### 4.3.2. Monotonicity of $\bar{D}\left(N\right)$

It is not difficult to conclude from Equation (13) that $\bar{D}\left(N\right)$ is a monotone decreasing function w.r.t the number of sensor nodes *N* by using Mathematical induction:

$$\begin{array}{cc} & \bar{D}\left(2\right)-\bar{D}\left(1\right)=\Gamma \left(0,1\right)e-2\Gamma \left(0,2\right)e^{2}<0;\\ & \bar{D}\left(3\right)-\bar{D}\left(2\right)=\Gamma \left(0,1\right)e-4\Gamma \left(0,2\right)e^{2}+3\Gamma \left(0,3\right)e^{3}<0; \end{array}$$

by applying the same method, it yields:

$$\begin{array}{cc} & \bar{D}\left(N\right)-\bar{D}\left(N-1\right)<0. \end{array}$$

#### 4.3.3. Monotonicity of the Weighted-Sum Tradeoff

Since the average AoI $\bar{\Delta }\left(N\right)$ monotonically increases w.r.t. *N*, and $\bar{D}\left(N\right)$ monotonically decreases w.r.t. *N*. The conclusion can be drawn that the weighted-sum is most time a unimodal function that decreases first and increases then, even it may fluctuate sometimes, we can still treat the first local optimum as global optimum since it saves system cost for deploying sensors. The monotonicity analysis paves the way for finding a sub-optimal number of sensor nodes which we will elaborate on in the next section.

## 5. Sub-Optimal Number of Sensor Nodes

In this section, we give a closed-form sub-optimal number of sensor nodes to achieve the best system timeliness and fidelity. By applying proper approximations on two metrics, a closed-form sub-optimal number of sensor nodes can be derived.

**Theorem** **1.** *The sub-optimal number of sensor nodes can be approximately given by:*

(21) $$\begin{array}{c} N^{*}=\arg\;\min\left\{f\left(\left\lfloor \tilde{N}\right\rfloor \right),f\left(\left\lceil \tilde{N}\right\rceil \right)\right\}, \end{array}$$

*where $\tilde{N}=\exp\left(-\frac{1}{\gamma_{0}}+\sqrt{\frac{2v(1-\alpha )}{3\alpha }}\right)$.*

**Proof of Theorem 1.**  Firstly, recall that $\bar{\Delta }\left(N\right)=\frac{3H_{N}}{2v}+\frac{G_{N}}{2vH_{N}}$, in fact, the latter part $\frac{G_{N}}{2vH_{N}}$ is small enough to omit, then we have $\bar{\Delta }\left(N\right)\approx \frac{3H_{N}}{2v}$. For MMSE, we try to use $1/𝔼[g+1/\gamma_{0}]$ to approach $\bar{D}\left(N\right)$. That is

(22) 1𝔼[g+1γ0]=1∫0∞(g+1γ0)p(g)dg=1∑i=1N(−1)i−1iN∫0∞ige−igdg+∫0∞iγ0e−igdg=(b)1HN+∑i=1N(−1)i−1iN1γ0=1HN+1γ0

where (b) follows from: ∑i=1N(−1)i−1iN∫0∞ige−igdg=HN.

Then, the objective problem can be further reformulated as:

(23) $$\begin{array}{c} f\left(N\right)=\frac{3\alpha H_{N}}{2v}+\frac{1-\alpha }{H_{N}+\frac{1}{\gamma_{0}}}. \end{array}$$

By searching for the zero-point of its first-order derivative and omit the unnecessary root, the sub-optimal number of sensor nodes can be figured out through

(24) $$\begin{array}{c} \frac{3\alpha }{2vN}-\frac{1-\alpha }{N(H_{N}^{2}+\frac{2H_{N}}{\gamma_{0}}+\frac{1}{\gamma_{0}^{2}})}=0. \end{array}$$

Noticing that $H_{N}\approx \ln N$, it yields

(25) $$\begin{array}{c} 3\alpha (\ln^{2}N+\frac{2\ln N}{\gamma_{0}}+\frac{1}{\gamma_{0}^{2}})-\left(1-\alpha \right)2v=0. \end{array}$$

After some manipulations, $\tilde{N}=\exp\left(-\frac{1}{\gamma_{0}}+\sqrt{\frac{2v(1-\alpha )}{3\alpha }}\right)$ can be calculated where another unnecessary root is omitted.

At last, by comparing $f(\left\lfloor \tilde{N}\right\rfloor )$ with $f(\left\lceil \tilde{N}\right\rceil )$, the solution $N^{*}$ can be obtained by choosing the integer resulting in smaller data freshness and fidelity weighted-sum tradeoff. □

One can conclude from Theorem 1 that the optimal number of sensor nodes $N^{*}$ is strongly affected by SNR $\gamma_{0}$, the weighting factor α and the arrival rate *v*.

## 6. Numerical Results

In this section, we firstly perform simulations to validate the correctness of theoretical results. After that, we investigate the effects of system parameters on the overall system freshness and fidelity. The realistic setup is shown in Table 1.

**Figure 3:**
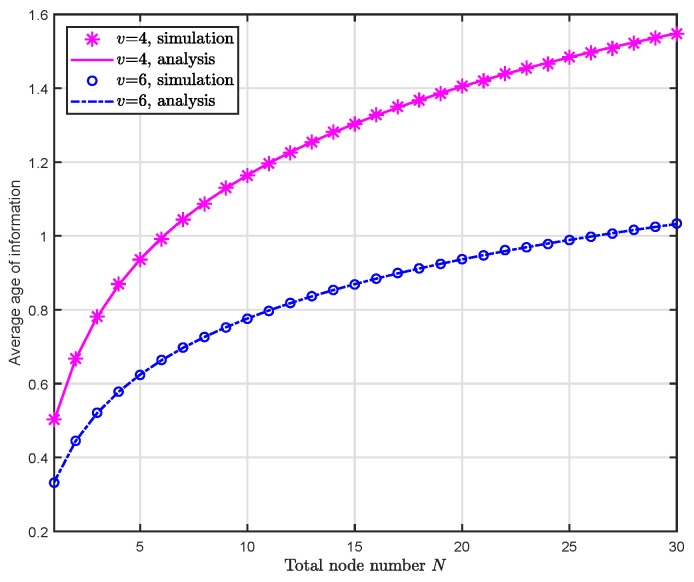
Simulation and analysis results of the average AoI $\bar{\Delta }\left(N\right)$ w.r.t. the number of sensor nodes *N* under different arrival rate *v*.

Figure 3 shows the simulation and analysis results of the average AoI $\bar{\Delta }\left(N\right)$ when the arrival rate of the updates are v=4 and v=6, i.e., in each time slot, there are 4 and 6 updates arriving at the FC respectively. As can be seen intuitively, the average AoI increases while the number of sensor nodes *N* grows. This accords with the analysis in Section 4.3.1. It is also validated that the average AoI is overall larger when the arrival rate *v* is smaller (cf. Equation (7)). Most importantly, by comparing the curves with the markers we can conclude that our analysis result on average AoI (cf. Lemma 1) is identical to the simulation result in which we invoked 100,000 groups of arrays obeying exponential distribution with parameter *v* to calculate the average AoI.

**Figure 4:**
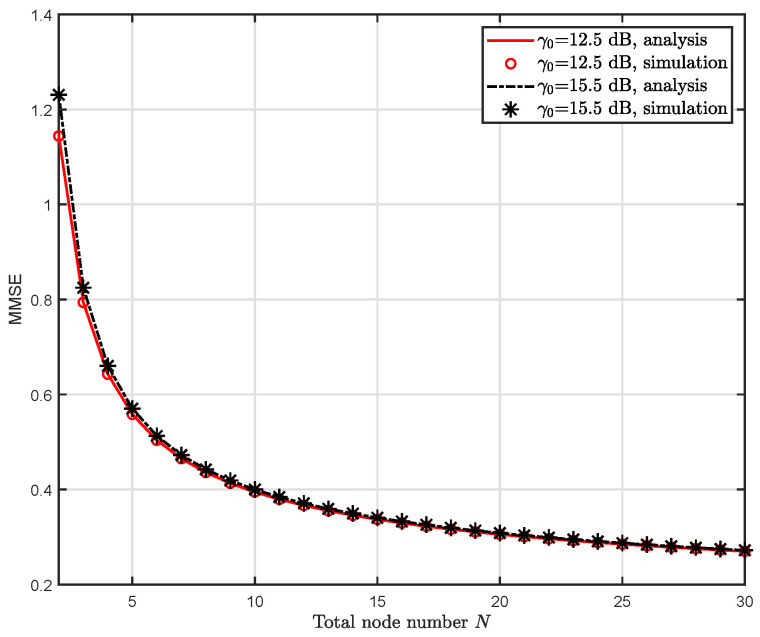
Simulation and analysis results of the average MMSE $\bar{D}\left(N\right)$ w.r.t. the number of sensor nodes *N* under different SNR $\gamma_{0}$.

Figure 4 shows the simulation and analysis results of the MMSE $\bar{D}\left(N\right)$. The realistic setup in this simulation is introduced as follows. The signal power $\sigma_{X}^{2}$ is set to 10 dBm (10 mW) and 13 dBm (20 mW) respectively, the channel noise power $\sigma_{Z}^{2}$ is $N_{0}B=-173\times 10^{6}$ dBm. For the path loss model, we adopt $35.5+37.6\mathrm{lg}(d_{k})$, where we assume the distance between the sensors and the FC $d_{k}$ is 100 m. Then we have the corresponding SNR $\gamma_{0}$ as 12.5 dB and 15.5 dB respectively. Firstly, it can be seen from the figure that our theoretic analysis on MMSE (Lemma 2) accords with the simulation in which we invoked 100,000 groups of random variables obeying $|h_{i}{|}^{2}∼\exp\left(1\right)$ and chose the largest among them as the received signal to further calculate the MMSE. Secondly, it is shown that the MMSE declines with the growth of sensor nodes number *N*, which also echoes with the analysis in Section 4.3.2.

**Figure 5:**
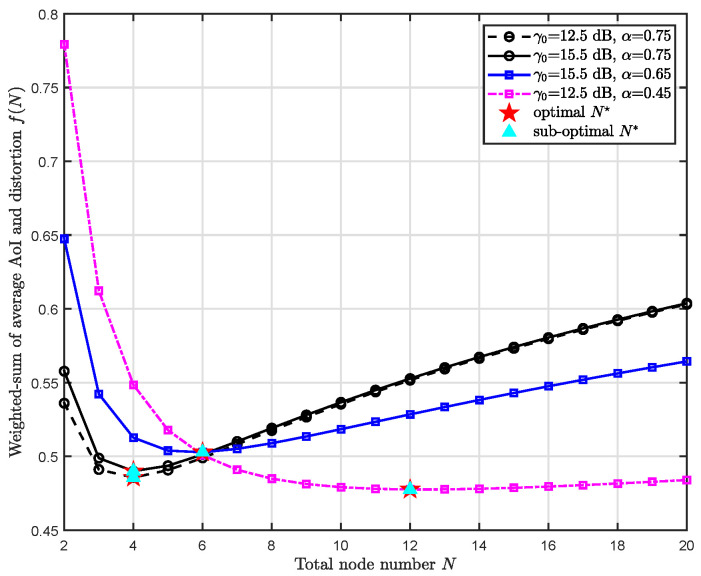
The weighted-sum of freshness and fidelity tradeoff f(N) w.r.t. the number of sensor nodes *N* under different SNR $\gamma_{0}$ and weighting factor α.

Figure 5 shows the varying trend of the data freshness-fidelity weighted-sum tradeoff f(N) with respect to the number of sensor nodes *N* under different SNR $\gamma_{0}$ and weighting factor α. The realistic setup of the system is the same as in Figure 4. In this case, we consider the same arrival rate as v=8 (eight updates arrive at the FC in each time slot). Overall, all curves well demonstrate the monotonicity of the weighted-sum tradeoff which decreases first and goes up then. Comparing circular dotted curve with a circular solid curve which differs in SNR $\gamma_{0}$, we can conclude that a smaller SNR $\gamma_{0}$ results in smaller MMSE and so is the tradeoff, whereas the optimal number of sensor nodes $N^{🟉}=4$ remains unchanged. Comparing the circular solid curve with the square solid curve, it is seen that a larger weighting factor α leads to a smaller optimal number of sensor nodes $N^{🟉}$. This can be ascribed to the fact that a larger weighting factor α means a larger AoI, hence the curve begins the upward trend earlier. By further reducing the weighting factor α to 0.45, the optimal number of sensor nodes becomes even larger, see the dotted square curve, reaching $N^{🟉}=12$. Most importantly, it is obvious that our proposed sub-optimal number of sensor nodes $N^{*}$ is identical to the optimal $N^{🟉}$. This well validates the effectiveness of our proposed sub-optimal number of sensor nodes.

**Figure 6:**
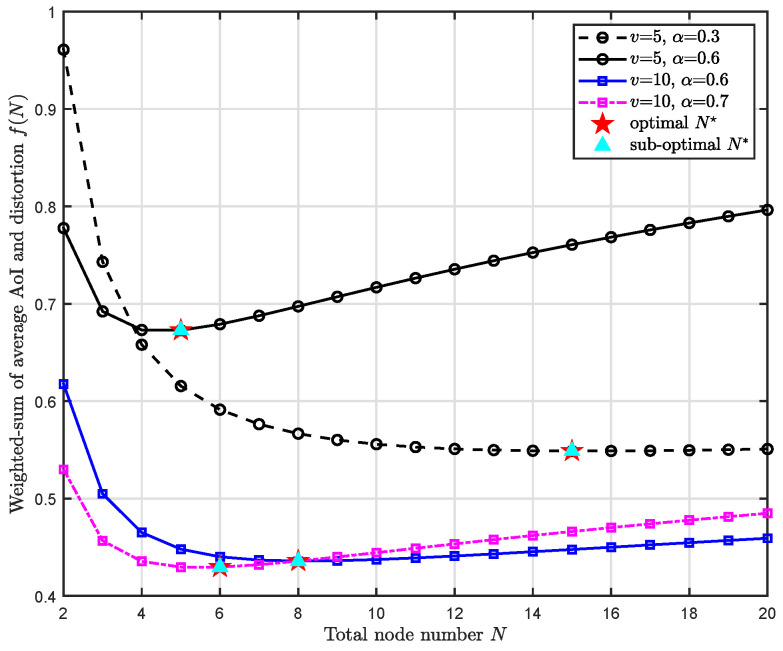
The weighted-sum of freshness and fidelity tradeoff f(N) w.r.t. the number of sensor nodes *N* under different and weighting factor α and arrival rate *v*.

Figure 6 depicts the relationship between the data freshness-fidelity weighted-sum tradeoff f(N) and the number of sensor nodes *N* under different weighting factor α and arrival rate *v*. In the simulation, the signal power $\sigma_{X}^{2}$ is fixed to 10 dBm (10 mW) and the noise power remains the same, which means the MMSE distortion remains the same. Observing the circular solid curve and the square solid curve, we can see that under the same weighting factor α=0.6, a larger arrival rate *v* causes a smaller tradeoff. However, the optimal number of sensor nodes $N^{🟉}$ can be comparatively larger: When v=10, $N^{🟉}=8$; When v=5, $N^{🟉}=5$. This is explainable since a larger arrival rate *v* leads to smaller AoI, which means the curve will start climbing later. The improvement on system overall freshness and fidelity brought by the proposed sub-optimal solution $N^{*}$ is also validated.

## 7. Conclusions

Motivated by the contradictory relationship between the freshness and fidelity of the received data, we studied the joint optimization of the two metrics by adjusting the number of sensor nodes. Explicit expressions of average AoI and MMSE were derived, based on which a closed-form sub-optimal solution was obtained via feasible approximations. Numerical results validate that our proposed sub-optimal number of sensor nodes is correct and can achieve the best data freshness and fidelity tradeoff with negligible errors.

## Figures and Tables

**Table 1:** Parameters.

System Parameter	Value
Single-sided noise spectral density $N_{0}$	−173 dBm/Hz
Available bandwidth $W_{\max}$	1 MHz
Path loss model $10\mathrm{lg}(\alpha_{k})$	$35.5+37.6\mathrm{lg}(d_{k})$ [27]
Maximum transmit power of sensor	13 dBm (20 mW) [27]

## References

[1] Sonehara M., Van Toai N., Sato T. (2016). Fundamental Study of Non-Contact Water Salinity Sensor by Using Electromagnetic Means for Seawater Desalination Plants. IEEE Trans. Magn..

[2] Lee S., Ko Y. (2009). Joint Selection Combining and Power Loading Transmission With Adaptive M-QAM in Multichannel System. IEEE Trans. Veh. Technol..

[3] Al-Suhail G.A., Lamahewa T.A., Kennedy R.A. Performance of dual-branch diversity receiver based SR-ARQ in Rayleigh fading channel. Proceedings of the 3rd International Conference on Signal Processing and Communication Systems.

[4] Rao B.D., Ming Y. (2003). Performance of maximal ratio transmission with two receive antennas. IEEE Trans. Commun..

[5] Young G., Sang W. (2005). Optimum selection combining for M-ary signals in frequency-nonselective fading channels. IEEE Trans. Commun..

[6] AL-Suhail G.A., Kennedy R.A. Performance of hybrid ARQ on dual-branch diversity receiver in Rayleigh fading channel. Proceedings of the 2010 4th International Conference on Signal Processing and Communication Systems.

[7] Kaul S., Yates R., Gruteser M. Real-time status: How often should one update?. Proceedings of the IEEE INFOCOM.

[8] Moltafet M., Leinonen M., Codreanu M. (2020). On the Age of Information in Multi-Source Queueing Models. IEEE Trans. Commun..

[9] Soysal A., Ulukus S. (2021). Age of Information in G/G/1/1 Systems: Age Expressions, Bounds, Special Cases, and Optimization. IEEE Trans. Inf. Theory.

[10] Zhang T., Chen S., Chen Z. (2021). Internet of Things: The Optimal Generation Rates under Preemption Strategy in a Multi-Source Queuing System. Entropy.

[11] Hu L., Chen Z., Dong Y., Jia Y., Liang L., Wang M. (2021). Status Update in IoT Networks: Age-of-Information Violation Probability and Optimal Update Rate. IEEE Internet Things J..

[12] Costa M., Codreanu M., Ephremides A. (2014). Age of information with packet management. Proc. IEEE Int. Symp. Inf. Theor..

[13] Yang H.H., Arafa A., Quek T.Q.S., Poor H.V. (2021). Optimizing Information Freshness in Wireless Networks: A Stochastic Geometry Approach. Proc. IEEE Trans. Mob. Comput..

[14] Hu L., Chen Z., Jia Y., Wang M., Quek T.Q.S. (2021). Asymptotically Optimal Arrival Rate for IoT Networks With AoI and Peak AoI Constraints. IEEE Commun. Lett..

[15] Dong Y., Chen Z., Liu S., Fan P., Ben Letaief K. (2020). Age-upon-decisions minimizing scheduling in Internet of Things: To be random or to be deterministic?. IEEE Internet Things J..

[16] Rajput K.P., Verma Y., Venkategowda N.K.D., Jagannatham A.K., Varshney P.K. (2021). Robust Linear Transceiver Designs for Vector Parameter Estimation in MIMO Wireless Sensor Networks Under CSI Uncertainty. IEEE Trans. Veh. Technol..

[17] Cheng X., Khanduri P., Chen B., Varshney P.K. (2021). Joint Collaboration and Compression Design for Distributed Sequential Estimation in a Wireless Sensor Network. IEEE Trans. Signal Process..

[18] Rajput K.P., Ahmed M.F., Venkategowda N.K.D., Jagannatham A.K., Sharma G., Hanzo L. (2021). Robust Decentralized and Distributed Estimation of a Correlated Parameter Vector in MIMO-OFDM Wireless Sensor Networks. IEEE Trans. Commun..

[19] Trihinas D., Pallis G., Dikaiakos M.D. (2021). Low-Cost Adaptive Monitoring Techniques for the Internet of Things. IEEE Trans. Serv. Comput..

[20] Bastopcu M., Ulukus S. (2021). Age of Information for Updates With Distortion: Constant and Age-Dependent Distortion Constraints. IEEE/ACM Trans. Netw..

[21] Arafa A., Seddik K.B.K.G., Poor H.V. (2021). Sample, Quantize, and Encode: Timely Estimation Over Noisy Channels. IEEE Trans. Commun..

[22] Roth S., Arafa A., Sezgin A., Poor H.V. (2021). Short Blocklength Process Monitoring and Scheduling: Resolution and Data Freshness. IEEE Trans. Wirel. Commun..

[23] Dong Y. (2020). Distributed Sensing With Orthogonal Multiple Access: To Code or not to Code?. IEEE Trans. Signal Process..

[24] Liu S., Kar S., Fardad M., Varshney P.K. (2016). Optimized Sensor Collaboration for Estimation of Temporally Correlated Parameters. IEEE Trans. Signal Process..

[25] Liu S., Chepuri S.P., Fardad M., Masazade E., Leus G., Varshney P.K. (2016). Sensor Selection for Estimation with Correlated Measurement Noise. IEEE Trans. Signal Process..

[26] Arnold B.B.C., Balakrishnan N., Nagaraja H.N. (2008). A First Course in Order Statistics.

[27] Study on Scenarios and Requirements for Next Generation Access Technologies, Technical Specification Group Radio Access Network, Release 14, Document 38.913, 3GPP, October 2016. https://portal.3gpp.org/desktopmodules/Specifications/SpecificationDetails.aspx?specificationId=2996.

